# UPLC-MS/MS Determination of Linezolid and Heme in Plasma of Infected Patients and Correlation Analysis

**DOI:** 10.1155/2021/6679076

**Published:** 2021-07-10

**Authors:** Yingying Wang, Xuemei Ye, Qin Lan, Xiaofang Ke, Lufeng Hu, Lichuan Hu

**Affiliations:** ^1^Department of Pharmacy, The First Affiliated Hospital of Wenzhou Medical University, Wenzhou 325000, China; ^2^Department of Pharmacy, Wenzhou Central Hospital, Wenzhou 325000, China

## Abstract

Linezolid can cause serious haematological toxicity, such as thrombocytopenia and aneamia. Heme, composed of iron and porphyrin, is an important component of hemoglobin. In order to investigate the relationship between the concentration of linezolid and heme in the plasma of infected patients, a UPLC-MS/MS method that can determine the concentrations of linezolid and heme simultaneously was developed and validated. A total of 96 healthy subjects and 81 infected patients, who received blood routine blood tests, were included and determined by the UPLC-MS/MS method. The results showed that the concentration of linezolid was 5.08 ± 3.46 *μ*g/mL in infected patients who were treated with linezolid. The heme in healthy subjects was 7.05 ± 8.68 *μ*g/mL, and it was significantly decreased to 0.88 ± 0.79 *μ*g/mL in infected patients (*P* < 0.01). Spearman correlation analysis showed that linezolid had a high negative correlation with platelet (PLT) (*R* = −0.309). Heme had a high positive correlation with hemoglobin (Hb) (*R* = 0.249) in healthy subjects and infected patients. The ROC analysis showed that heme had diagnostic value to distinguish low Hb (110 g/L). In conclusion, there was a positive correlation between heme and Hb, and this correlation was also observed in infected patients. A high concentration of linezolid was inclined to decrease PLT. Monitoring of heme and linezolid helps in the early diagnose of low Hb and PLT.

## 1. Background

Linezolid, the first new class of oxazolidinones antibiotics [1], is used for the treatment of multidrug-resistant gram-positive pathogens, including vancomycin-resistant enterococci (VRE), methicillin-resistant staphylococcus aureus (MRSA), and several anaerobes [2]. Moreover, linezolid has been recommended for the treatment of patients with multidrug-resistant (MDR) or extensively drug-resistant (XDR) tuberculosis [3]. However, linezolid causes high incidences of severe side effects, such as haematological toxicity, hyperlactatemia, metabolic acidosis, gastrointestinal disturbances, and peripheral neuropathy [4]. The most common is haematological toxicity, with the incidence of thrombocytopenia and anemia at 32%-45.8% and 25%, respectively [5]. Hanai et al. reported that thrombocytopenia and anemia developed in 48.4% and 10.4% of patients during linezolid therapy [6].

Heme is an essential substance, which can be synthesized by bacteria and human cells [7]. It is composed of iron and porphyrin and considered as an important component of many proteins, such as oxidase protein [8, 9], cytochrome protein [10], hemoglobin, and cytochrome P450 protein [11]. Moreover, heme is essential for electron transport [12], metabolism of oxygen molecules and diatomic gases [13], and various redox reactions [14], and thus, plays important roles in a number of metabolic pathways of organisms.

Some studies have been carried out to investigate the toxic mechanisms of linezolid, such as mitochondrial ribosome inhibition [15], tissue-dependent mitotoxicity [4], deficiency of mitochondria encoded proteins [16], and similarities between human and bacterial ribosomes [17]. Those studies revealed part of the reason for adverse hematologic and peripheral nervous side effects, however, few of them focused on the heme, and the exact mechanism of its haematological toxicity is still unclear. Considering the important physiological function of heme, this study is aimed at developing a UPLC-MS/MS method to determine the heme and linezolid levels in infected patients and to investigate the correlation between them.

## 2. Methods

### 2.1. Ethics Statement and Subjects

This study was approved by the Ethics Committee of the First Affiliated Hospital of Wenzhou Medical University (2020-zz-219) and conducted in accordance with the Declaration of Helsinki. Data from all infected patients and healthy subjects were analyzed anonymously and securely. All data are for the use of investigators only.

### 2.2. Reagents and Instruments

Heme and linezolid were purchased from Sigma-Aldrich (Lewis, USA, lot: J0804A) and TRC (Toronto, Canada, lot: L466500). Fluconazole (purity > 98%, lot: 481850) was purchased from national institutes for food and drug control (Peking, China). Acetonitrile, methanol, and formic acid were purchased from Merck Company (Darmstadt, Germany), and all reagents were at the HPLC grade. Ultrapure water (resistance > 18 m*Ω*) was prepared by a Millipore Milli-Q purification system (Bedford, USA).

The ACQUITY UPLC system consists of a Binary Solvent Manager (BSM) and a Sample Manager with a Flow-Through Needle (SM-FTN). Heme and linezolid were analyzed using the Xevo TQ-S Micro triple quadrupole mass spectrometer (Waters Corporation, USA) equipped with an electrospray ion source (ESI).

### 2.3. UPLC-MS/MS Determination of Linezolid and Heme

Chromatographic separation of linezolid, heme, and fluconazole (IS) was conducted at BEH C18 column (2.1 mm × 100 mm, 1.7 *μ*m Waters Corporation) at 40°C. The mobile phase consists of 0.1% formic acid water (A), and acetonitrile (B) was used in gradient elution as follows: (Tmin/acetonitrile): 0.0-0.5/20%, 0.5-0.8/80%, 0.8-2.5/80%, and 2.5-3.0/20%. The flow rate was set at 0.3 mL/min.

Linezolid, heme, and IS were detected by multiple reaction monitoring (MRM) mode. The UPLC-MS/MS conditions are listed in Table 1. The desolvation temperature was 600°C, cone gas flow was 150 L/hr, and desolvation gas flow was 1000 L/hr. The injection volume was 0.5 *μ*L. All UPLC-MS/MS data were collected and processed by Masslynx 4.1 software (Waters Corp, MA, USA).

### 2.4. Calibration Curve and Sample Preparation

The stock solution of heme was prepared in alkaline solution at a concentration of 1.00 mg/mL (1 mL water added with 5 *μ*L saturated sodium hydroxide), and linezolid was prepared in methanol at 1.00 mg/mL. The calibration standards were prepared by spiking 5 *μ*L mixed standard solutions of linezolid and heme into 45 *μ*L plasmas. The added concentrations of standard curve samples were 0.5, 1, 2, 4, 8, 16, and 32 *μ*g/mL.

The 50 *μ*L plasma samples were precipitated by 200 *μ*L of 1% formic acid-acetonitrile, supplemented with 0.05 *μ*g/mL of IS. Then, the mixture was vortexed for 0.2 min, centrifuged at 15000 rpm for 5 min, and 0.5 *μ*L supernatant was injected into the UPLC-MS/MS system for analysis.

### 2.5. Method Validation

Precision, precision, recovery, matrix effect, and stability of the method were verified with 2, 4, and 8 *μ*g/mL quality control samples. Diurnal precision of heme and linezolid was assessed at three quality control levels, repeated three times a day, and for three consecutive days.

The extraction recovery was evaluated by comparing the peak area of heme in pure standard solution at the same concentration. The matrix effect was investigated by comparing the peak area of heme with the same concentration in the extracted samples under three quality control levels. The stability of the three QC samples was tested at 2 h, 4 h, and 24 h at room temperature.

### 2.6. Infected Patients and Healthy Subjects

The subjects involved in this study were infected patients and healthy subjects from the First Affiliated Hospital of Wenzhou Medical University. All patients underwent regular clinical biochemical examinations, including blood routine test (BRT) and liver and kidney function examination. After completing the routine blood test, the blood samples of healthy subjects will be collected and stored at -80°C for heme detection. Blood samples were collected for the determination of linezolid and heme in infected persons receiving linezolid treatment.

The BRT and biochemical indices were analyzed with Beckman AU5800 biochemical measurement and Sysmex XE-2100 automated hematology analyzer. Linezolid and heme were determined by the developed UPLC-MS/MS method.

### 2.7. Statistical Analysis

The differences of BRT between infected patients and healthy subjects were analyzed by using independent samples test. The relationship between linezolid and heme and BRT was analyzed by Spearman's bivariate correlation. The receiver operating characteristic curve (ROC) was used to evaluate the diagnostic value of linezolid and heme. All statistical differences were analyzed using SPSS software 17.

## 3. Results

### 3.1. UPLC-MS/MS Determination of Heme and Linezolid

**Figure 1:**
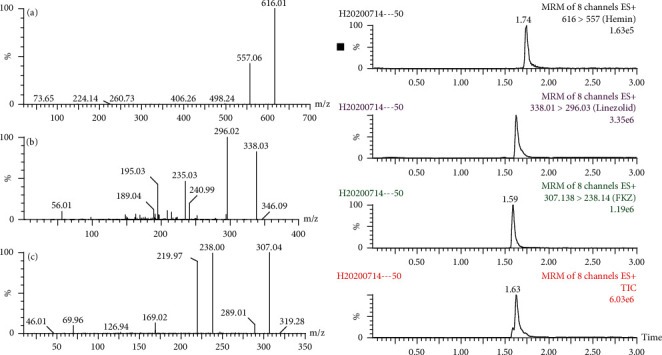
UPLC-MS/MS mass spectrogram and chromatogram of heme (a), linezolid (b), and IS (c).

According to the optimized UPLC and mass conditions, the typical mass spectrums and UPLC-MS/MS chromatograms are shown in Figure 1. Heme, linezolid, and IS were eluted at 1.74 min, 1.63 min, and 1.59 min, respectively. No endogenous compounds interfere with heme, linezolid, and IS in positive ion mode.

The calibration curve of heme and linezolid showed a good linear relationship in the concentration range of 0.5-32 *μ*g/mL. The regression equation and coefficient of heme are as follows: *y* = 0.1727*x* − 0.1132, *R*^2^ = 0.9953; and those of linezolid are as follows: *y* = 15.938*x* − 1.1024, *R*^2^ = 0.9983. According to the signal-to-noise ratio (S/N) of 3, the detection limit of heme was 1.3 ng/mL, and that of Linezolid was 0.5 ng/mL.

The relative standard deviation (RSD) of intraday and interday precision of heme and linezolid was less than 15%, the extraction recovery was over 75%, and the matrix effect was over 83%. The results are shown in Table 2. The stability RSD of both heme and linezolid at room temperature was no more than 15%.

### 3.2. Clinical Indexes of Infected Patients and Subjects

A total of 81 infected patients (64 males, 17 females) and 96 healthy subjects (35 males, 61 females) were included, with average ages of 61.99 ± 17.21 and 43.89 ± 11.48 years old, respectively. Linezolid concentrations, BRT, and renal and liver function tests of a total of 107 infected patients were recorded. The differences in BRT and renal and liver function tests between healthy subjects and infected patients are listed in Tables 3 and 4. The results showed that the WBC, percentage of neutrophil, and the absolute value of neutrophil and monocytes in infected patients were higher than those of healthy subjects (*P* < 0.05), while the RBC, Hb, and HCT in infected patients were lower (*P* < 0.05). Renal and liver function tests showed that indirect bilirubin, total protein, and albumin decreased, while direct bilirubin, ALT, AST, ALP, *γ*-GT, and BUN increased in infected patients.

### 3.3. Heme Level in Healthy Subjects and Infected Patient

**Figure 2:**
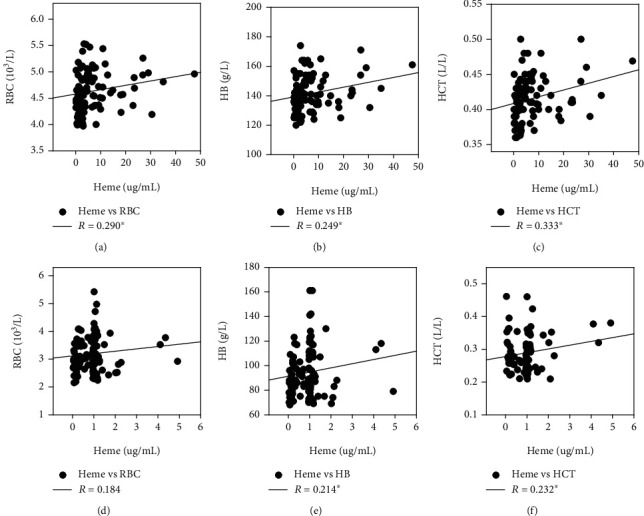
Correlation of heme with RBC, Hb, and HCT in healthy subjects (ABC) and infected patients (DEF). *R*: correlation coefficient of Spearman's analysis. ∗*P* < 0.05.

**Figure 3:**
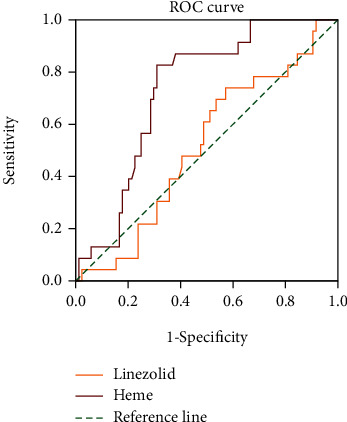
The ROC analysis of heme (0.731) and linezolid (0.515) in infected patients.

Based on the developed UPLC-MS/MS method, the mean concentration of heme in healthy subjects was 7.05 ± 8.68 *μ*g/mL, while the mean heme concentration in infected patients was significantly decreased to 0.88 ± 0.79 *μ*g/mL (*P* < 0.01). Spearman correlation analysis showed that heme had a high positive correlation with RBC (*R* = 0.290), Hb (*R* = 0.249), and HCT (*R* = 0.333) in healthy subjects. In the infected patients, heme was highly positively correlated with Hb and HCT (*R* = 0.214, *P* = 0.027). The correlation analysis between heme BRT indexes is shown in Figure 2 and Supplementary Table 1. Further ROC analysis (Figure 3) showed that heme was more valuable than linezolid in the diagnosis of low HB (110 g/L).

### 3.4. Correlation Analysis of Linezolid and Heme in Infected Patients

**Figure 4:**
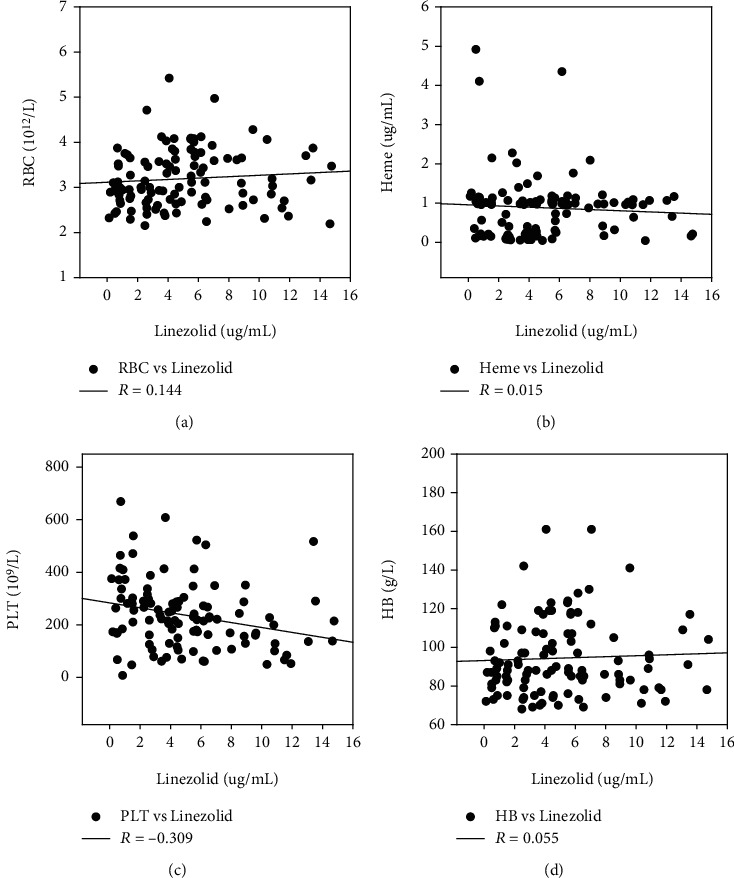
Correlation of linezolid with RBC, Hb, PLT, and heme in infected patients (DEF). *R*: correlation coefficient of Spearman's analysis. ∗*P* < 0.05.

Based on the developed UPLC-MS/MS method, we found that the mean serum concentration of linezolid was 5.08 ± 3.46 *μ*g/mL. Spearman correlation analysis showed that linezolid was negatively correlated with WBC, neutrophil, monocytes, and PLT (*R* = −0309) and positively correlated with eosinophils and lymphocytes. However, there was no correlation between RBC, HB, and heme. The correlations between linezolid and RBC, Hb, PLT, heme, and other indices are shown in Figure 4 and Supplementary Table 2.

## 4. Discussion

So far, it has been reported that spectral deconvolution [18] and high-performance liquid chromatography diode array spectrophotometry [19] have been used to determine heme. In terms of HPLC-MS/MS, Fyrestam and Ostman [20] developed a liquid-liquid extraction method for the determination of heme in microorganisms by HPLC-MS/MS. Whiteaker et al. developed matrix-assisted laser desorption/ionization time-of-flight mass spectrometry (MALDI-TOFMS) for the determination of heme (ferriprotoporphyrin IX) in bacillus spores [21]. However, these methods are rarely used for the determination of heme in plasma.

Linezolid can be determined by HPLC-UV [22], UHPLC-PDA [23], quinone-based fluorophores [24], and LC-MS-MS [25]. HPLC-UV and LC-MS-MS are two widely used methods in clinical practice. Compared with HPLC, LC-MS-MS is simple and sensitive and specific. Moreover, LC-MS-MS can simultaneously determine multiple substances. So far, although several methods for the determination of linezolid in plasma have been reported, most of them are about simultaneous determination with other drugs, such as meropenem and theophyllin [26], piperacillin and teicoplanin [27], and daptomycin and tedizolid [28]. In this study, an UPLC-MS/MS method for simultaneous determination of heme and linezolid in plasma was developed for the first time. The results of method validation showed that our method was fast, convenient, and precise, and can be used to determine the heme and linezolid levels in patients.

In this study, 81 infected patients were included in this study. The blood, sputum, and urine culture tests showed that the main infectious bacteria were staphylococcus aureus, escherichia coli, and acinetobacter baumannii. The BRT, renal, and liver function tests showed that WBC, percentage of neutrophil, direct bilirubin, ALT, AST, ALP, and BUN increased. It indicated that there were obvious infected states in those infected patients.

So far, most studies have investigated the heme oxygenase-1 in patients with various infections [29, 30], such as enterohemorrhagic Escherichia coli [31] and Mannheimia haemolytica infection [32], but few studies have focused on heme. In order to evaluate the heme level in infected patients, we included healthy subjects and infected patients. The Spearman correlation analysis showed that heme was related to WBC (0.221), RBC (0.290), and HB (0.249) in healthy subjects and had statistics difference (*P* < 0.05). While, in infected patients, only HB was related to heme (0.214, *P* < 0.05). It is indicated that there is a stable connection between heme and Hb, which does not change with infection.

It has been widely acknowledged that there is a significant correlation between the plasma concentration of linezolid and hemotoxicity in infected patients [33]. Even in healthy volunteers, linezolid intravenously reduced Hb and RBC levels [34]. Therefore, it is necessary to determine the plasma concentration of linezolid in infected patients. Our results showed that the plasma concentration of linezolid, which ranged from 0.5 to 14.7 *μ*g/mL, was highly correlated with PLT, but not with Hb, RBC, and heme. Dou et al. reported when AUC_24_ > 243 mg · h/L or *C*_min_ ≥ 6.3 mg/L, the probability of thrombocytopenia was >50% [35]. These results indicated that PLT was more sensitive to the serum concentration of linezolid than RBC and HB. In other words, the higher the concentration of linezolid, the lower the PLT level.

## 5. Conclusions

A sensitive, reliable, and accurate UPLC-MS/MS for simultaneous determination of heme and linezolid was developed. The positive correlation between heme and Hb was stable and did not change with different infections. Compared with healthy subjects, the heme level was significantly decreased in infected patients (*P* < 0.01). Linezolid decreased PLT but not RBC and HB. Monitoring heme and linezolid can help in the early diagnose of low Hb and PLT.

## Figures and Tables

**Table 1:** UPLC-MS/MS conditions of linezolid, heme, and (IS).

Compound	Ion mode	Parent ion (m/z)	Daughter ion (m/z)	Cone (V)	Collision
Linezolid	Positive	338.03	296.02	10	15
Heme	Positive	616.0	557.0	10	35
IS	Positive	307.1	238.1	6	16

**Table 2:** The precision, extraction recovery, and matrix effects of linezolid and heme.

Compound	*μ*g/mL	Precision (RSD)		Extraction recovery (mean ± SD, %)	Matrix effects (mean ± SD, %)
		Intraday	Interday		
	3	9.10	11.22	86.45 ± 12.47	83.99 ± 11.24
Linezolid	6	8.20	14.53	85.12 ± 11.95	77.02 ± 6.78
	12	2.19	12.53	75.37 ± 7.64	75.31 ± 7.01
	3	4.32	5.01	77.46 ± 1.86	98.76 ± 12.06

Heme	6	3.67	3.31	78.83 ± 6.40	95.40 ± 9.61
	12	1.72	3.76	77.30 ± 8.47	83.54 ± 6.48

**Table 3:** BRT indices of healthy subjects and infected patients (mean ± SD).

Indices	Healthy	Patient	*P*
White blood cell (WBC)	5.88 ± 1.30	9.85 ± 5.99	<0.001
Percentage of neutrophil	0.55 ± 0.07	0.74 ± 0.14	<0.001
Percentage of eosinophils	0.02 ± 0.01	0.02 ± 0.04	0.672
Percentage of basophils	0.0015 ± 0.0018	0.0013 ± 0.0018	0.504
Percentage of monocytes	0.07 ± 0.06	0.07 ± 0.04	0.52
Percentage of lymphocytes	0.36 ± 0.08	0.17 ± 0.11	<0.001
Absolute value of eosinophils	0.15 ± 0.26	0.19 ± 0.39	0.468
Absolute value of neutrophil	3.24 ± 0.99	7.82 ± 5.75	<0.001
Absolute value of monocytes	0.40 ± 0.13	0.61 ± 0.39	<0.001
Absolute value of lymphocyte	2.08 ± 0.53	1.32 ± 0.60	<0.001
Absolute value of basophils	0.01 ± 0.02	0.01 ± 0.02	0.182
Red blood cell (RBC)	4.64 ± 0.38	3.19 ± 0.63	<0.001
Hemoglobin (Hb)	141.57 ± 12.10	94.40 ± 19.56	<0.001
Hematocrit (HCT)	0.41 ± 0.05	0.28 ± 0.05	<0.001
Mean corpuscular volume	89.55 ± 3.02	89.11 ± 7.70	0.598
Mean hemoglobin	30.65 ± 1.40	29.67 ± 2.45	0.001
Mean hemoglobin concentration	334.76 ± 44.89	333.42 ± 15.97	0.773
RBC volume distribution width	12.83 ± 0.63	15.07 ± 2.48	<0.001
SD value of RBC volume distribution	84.21 ± 414.70	48.56 ± 9.40	0.375
Platelet (PLT)	237.65 ± 47.91	237.06 ± 128.52	0.966
Thrombocytocrit	0.26 ± 0.05	0.25 ± 0.12	0.800
Mean platelet volume	10.89 ± 0.85	10.70 ± 1.30	0.226
SD value of platelet distribution	13.18 ± 1.88	13.16 ± 3.36	0.950
Large platelet ratio	32.13 ± 7.18	30.31 ± 10.26	0.154

**Table 4:** hepatic and renal function indices of healthy subjects and infected patients (mean ± SD).

Indices	Healthy	Patient	*P*
Total bilirubin	12.20 ± 3.48	19.66 ± 37.72	0.057
Direct bilirubin	3.98 ± 1.10	13.43 ± 34.67	0.009
Indirect bilirubin	8.32 ± 2.61	6.20 ± 4.23	<0.001
Total protein	74.92 ± 3.87	62.82 ± 8.72	<0.001
Albumin	45.21 ± 2.52	32.16 ± 6.01	<0.001
Globulin	29.73 ± 3.50	30.66 ± 7.11	0.269
Alanine transaminase (ALT)	17.03 ± 7.86	41.39 ± 57.53	<0.001
Aspartate transaminase (AST)	20.97 ± 4.35	73.79 ± 244.11	0.035
Alkaline phosphatase (ALP)	71.28 ± 20.25	141.82 ± 111.15	<0.001
*γ*-Glutamyltransferase (*γ*-GT)	21.17 ± 10.00	81.40 ± 69.26	<0.001
Blood urea nitrogen (BUN)	4.94 ± 1.20	9.77 ± 9.31	<0.001
Creatinine	65.63 ± 12.51	137.97 ± 174.90	<0.001
Uric acid	302.91 ± 61.10	303.15 ± 167.53	0.990

## Data Availability

The datasets generated and/or analyzed during the current study are not publicly available but are available from the corresponding author on reasonable request.

## Supplementary Materials

Supplementary Materials Supplementary material included Supplementary Table 1, Table 2, and ethical approval documents.
