# Supplementary material for: UPLC-MS/MS Determination of Linezolid and Heme in Plasma of Infected Patients and Correlation Analysis
Source: Biomed Res Int. 2021 Jul 10;2021:6679076. doi: 10.1155/2021/6679076 (PMC8289587; doi:10.1155/2021/6679076)
Supplement: Supplementary Materials — Supplementary material included Supplementary Table 1, Table 2, and ethical approval documents. [file 6679076.f1.docx]

**Supplement Tables**

Supplement table 1 The Spearman's correlation analysis of heme with index BRT in healthy and infected patients

| Indices | | | healthy (n=96) | P | Patients  (n=107) | P |
| --- | --- | --- | --- | --- | --- | --- |
| White blood cell | |  | 0.221* | 0.03 | 0.078 | 0.427 |
| Percentage of Neutrophil | | | 0.019 | 0.851 | 0.127 | 0.196 |
| Percentage of eosinophils | | | 0.090 | 0.384 | -0.124 | 0.207 |
| Percentage of basophils | | | -0.169 | 0.099 | -0.222* | 0.022 |
| Percentage of monocytes | | | -0.012 | 0.908 | -0.054 | 0.579 |
| Percentage of lymphocytes | | | -0.015 | 0.881 | -0.114 | 0.244 |
| Absolute value of eosinophils | | | 0.106 | 0.305 | -0.128 | 0.200 |
| Absolute value of neutrophil | | | 0.194 | 0.059 | 0.112 | 0.265 |
| Absolute value of monocytes | | | 0.226* | 0.027 | 0.002 | 0.984 |
| Absolute value of lymphocyte | | | 0.165 | 0.107 | -0.182 | 0.067 |
| Absolute value of basophils | | | -0.103 | 0.318 | -0.224* | 0.024 |
| Red blood cell | |  | 0.290** | 0.004 | 0.184 | 0.058 |
| Hemoglobin | |  | 0.249* | 0.015 | 0.214* | 0.027 |
| Hematocrit | |  | 0.333** | 0.001 | 0.232* | 0.016 |
| Mean corpuscular volume | | | 0.056 | 0.591 | 0.08 | 0.41 |
| Mean hemoglobin | |  | -0.083 | 0.42 | 0.133 | 0.171 |
| Mean hemoglobin concentration | | | -0.133 | 0.196 | 0.03 | 0.756 |
| RBC volume distribution width | | | 0.136 | 0.187 | -0.163 | 0.093 |
| SD value of RBC volume distribution | | | 0.139 | 0.176 | -0.048 | 0.623 |
| Platelet |  |  | 0.099 | 0.337 | 0.135 | 0.166 |
| Thrombocytocrit | |  | 0.157 | 0.126 | 0.098 | 0.334 |
| Mean platelet volume | | | 0.095 | 0.359 | -0.137 | 0.176 |
| SD value of platelet distribution | | | 0.035 | 0.732 | -0.136 | 0.179 |
| Large platelet ratio | | | 0.085 | 0.411 | -0.146 | 0.149 |

Supplement table 2 The Spearman's correlation analysis of linezolid with heme and BRT index in infected patients

| Index | Coefficient | P |
| --- | --- | --- |
| heme | 0.015 | 0.877 |
| white blood cell | -0.296** | 0.002 |
| Percentage of neutral particles | -0.207* | 0.034 |
| Percentage of eosinophils | 0.251** | 0.01 |
| Percentage of basophils | -0.038 | 0.702 |
| Single core percentage | -0.059 | 0.548 |
| Percentage of lymph | 0.226* | 0.02 |
| Absolute value of eosinophils | 0.124 | 0.214 |
| Absolute value of neutral particle | -0.313** | 0.001 |
| Single core absolute value | -0.340** | <0.001 |
| Absolute value of lymph | 0.030 | 0.764 |
| Absolute value of basophils | -0.142 | 0.156 |
| Red blood cell | 0.144 | 0.138 |
| hemoglobin | 0.055 | 0.577 |
| Hematocrit | 0.013 | 0.892 |
| Mean corpuscular volume | -0.183 | 0.059 |
| Mean hemoglobin | -0.057 | 0.562 |
| Mean hemoglobin concentration | 0.181 | 0.062 |
| RBC volume distribution width | -0.134 | 0.167 |
| SD value of RBC volume distribution | -0.216* | 0.025 |
| Platelet | -0.309** | 0.001 |
| Thromboplastin | -0.301** | 0.002 |
| Mean platelet volume | 0.127 | 0.211 |
| SD value of platelet distribution | 0.147 | 0.147 |
| Large platelet ratio | 0.121 | 0.234 |
